# Diagnosing granulomatous disease during appendectomy

**DOI:** 10.1002/ccr3.5074

**Published:** 2021-11-16

**Authors:** Atilla Şenaylı

**Affiliations:** ^1^ Faculty of Medicine Pediatric Surgery Department T.C. Yozgat Bozok University Yozgat Turkey

**Keywords:** Appendicitis, case report, Children, Granulomatous, Surgery

## Abstract

Difficulties during surgery are uncommon situations in appendectomy. For granulomatous appendicitis, literature is insufficient about surgical findings. The procedure of a 17‐year‐old male patient was a struggle due to adhesions. I thought a surgeon could expect granulomatous diseases by evaluating the macroscopic appearance of the appendix during surgical procedure.

## Graphical abstract

Surgeons must be investigative about the feature of inflammed appendix for the possibility of uncommon findings that can reveal extraordinary disease such as granulomatous.

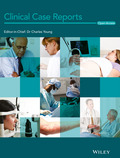

## 1 INTRODUCTION

Appendicitis is a disease that has been studied extensively in both adult and pediatric age groups. Despite the vast knowledge, this disease still confronts surgeons with surprising developments in terms of surgical procedures and clinical course.^1^, ^2^, ^3^, ^4^

Granulomatous diseases are found only in 0.1–2% of cases of appendicitis.^5^, ^6^, ^7^ The general characteristic of these diseases is that pathology results of the causative diseases are frequently reported as "not fully determined". It is stated that this is proper especially for diseases such as *Tuberculosis*, *Yersinia*, Sarcoidosis, and Crohn's Disease (CD).^7^

For the case, *Tuberculosis*, *Yersinia*, Sarcoidosis, *Actinomycosis*, and CD were evaluated among the prominent granulomatous diseases for the possible macroscopic findings to alert the surgeon for right diagnosis.

This case report aimed to investigate whether the surgeon can recognize the possibility of granulomatous disease in cases where appendectomy is problematic and difficult.

### 1.1 Case Presentation

A 17‐year‐old male patient was admitted to our hospital with nausea and right lower quadrant abdominal pain. He had no fever and other gastrointestinal complaints. It was learned that a similar abdominal pain complaint resolved in a short time without using medication three weeks ago. When asked in detail, it was found that carbohydrate‐weighted dietary preference (fried potatoes, pasta) contained approximately 75–80% of his total diet. There was no history of illness or surgery other than treatment for the hearing problem. There was no other disease in the family except for diabetes mellitus in her mother.

At laboratory, leukocyte count 14.9 x 103 / μL (N: 4–10), neutrophil count 74% (N: 50–70), lymphocyte 17.8% (N: 20–40), C‐RP 148.60 mg / L (N: 0–5), Sedimentation 99 (N: 0–20) were detected. Other blood counts and blood biochemistry examinations were normal. Although the physical examination findings were similar to appendicitis, ultrasonography (USG) was performed to evaluate other possible non‐surgical diseases, due to his good general condition and the absence of symptoms and signs such as fever, vomiting, dehydration, and anorexia. In the USG, the appendix could not be identified separately from the ascending colon structures, but it was stated that it could be in retrocaecal recess. An abscess containing septae in 40 x 35 mm size was detected. It was stated that there might be inflammation in the surrounding mesentery and intestinal tissues. Thereupon, laparoscopic exploration and appendectomy were planned under emergency conditions.

In laparoscopy, it was found that the appendix was firmly attached to the cecum in the cranial direction. There was no rolling‐up omentum. The borders of the appendix with the cecum could be distinguished. Inflammation was observed, but there was no congestion, increased vascularity and hyperemic appearance as would be expected in bacterial infections. The appearance of the fluid around the appendix was mostly like lymphatic extravasation. The tissue was concrete and abscess fluctuation was not present with forceps. As the thickness of the appendix was 1.5–2 cm, it was thought that there might be phlegmone. When the appendix could not be released from the cecum with laparoscopy forceps, the operation was converted to laparotomy. In laparotomy, we faced that appendix had an intense adhesion to the cecum. The dissections were similar to those of tissues with chronic fibrotic reaction, and the appendix was excised with difficulty. Ileum, cecum and ascending colon were in normal appearance on inspection and palpation. A penrose drain was placed in the surgical area and the procedure was ended. He was discharged 1 week after broad‐spectrum antibiotic therapy and routine clinical care. The appendix was defined as granulomatous disease, and Crohn's Disease (CD) was the final diagnose. He was healthy after 1‐year follow up. Recurrence of the disease was not seen.

## 2 DISCUSSION

Majority of the inflammations of appendectomy were described as in an ordinary range in the literature, and a standard course of the clinical process was occasionally defined.^8^, ^9^, ^10^, ^11^ For the macroscopic studies, in most of the available data, appendicular inflammation is classified as "normal", "inflamed", "phlegmone", "gangrenous", or "perforated", and therefore only the severity of the inflammation is taken into account but there are little descriptions in details.^12^, ^13^, ^14^, ^15^, ^16^, ^17^ For example, in the study of Pham et al., surgeons defined inflammations they saw, and pathology evaluations were compared with these macroscopic examinations. Eighty‐three percent of cases were compatible according to comparison of inflammation appearance in the specimen evaluation with the evaluation during surgery.^14^ Nevertheless, it is not clear in the study that if the surgeons define an inflammation as a granulomatous appendicitis. This was the fact for the rest of 17% evaluations in the same study.^14^ Similar situation is almost factual in other studies.^12^, ^13^

As, *Tuberculosi*s, *Yersinia*, Sarcoidoz, *Actimomycosis* and CD were evaluated for differential diagnosis during pathology examination; I decided to investigate the literature for these diseases for macroscopic appearance. Also appearance of the patients’ appendix was compared with these findings.

Andrews, described appearance of four patients in 1901, and defined the presence of "nodules", "cecal inflammation" and "agglomeration" in cases of appendicitis due to T*uberculosis*.^1^ The author stated macroscopically that it resembles carcinoma or look like an old appendicular abscess.^1^ He even indicated that existing adhesions caused problems leading to ileostomy.^1^ Such a severe surgical picture is not mentioned in contemporary articles. It has been stated in some studies that obtaining a “cottage‐cheese” appearance through the tissue during appendectomy may be evidence of tuberculosis.^5^, ^18^ In another article, *Tuberculous* appendicitis was reported as diffuse inflammatory mass and abscess.^19^ All these cases with *Tuberculosis* appendicitis were operated without obvious problem.^20^

Apart from the earlier publications, it can be concluded that there is no significant adhesion in cases of *Tuberculosis* appendicitis. In our case, condensed adhesion was detected with the cecum, which is not mentioned in the *Tuberculosis* articles examined. However, data is limited in literature, and more data is needed on this topic.

Although *Yersinia* infections are always recognized for the differential diagnosis of appendicitis, there were a few patients operated on for *Yersinia* appendicitis. This may be because of unspecific characteristic of *Yersinia* in appendicitis formation. In only one case of *Yersinia*, it was reported that there was thickening in the proximal part of the appendix and a resection‐ileocolic anastomosis was performed due to the thickening of the cecum and ileum.^21^ For this reason, the surgeon can expect not to see significant fibrosis and adhesions in appendicitis caused by *Yersinia*. However, if there is more data on this subject, an effective decision can be made.

Information about the surgical appearance of appendicitis cases with sarcoidosis is even more limited. In one study about sarcoidosis stated that appendix was edematous and swollen.^22^ In the article of Erra et al., it was stated that the patient, who was operated for sarcoidosis colitis, had 5 cm stenosis in the cecum and ascending colon along with many micro nodules in the peritoneum and accompanying lymphadenopathies, and it was stated that the appendix expanded up to 4 cm.^23^ The available information incompletely reminds us of the adhesions we see in our patient. However, it should be forgotten that sarcoidosis appendicitis alone is extremely rare.

In cases of *Actinomycosis*, the diameter of the appendix can progress with the formation of abscess with a diameter of 2 to 4 cm and omental encirclement can also be seen.^24^, ^25^ *Actinomycosis* appendicitis that reaches this thickness is usually perforated, but it can be detected rarely without perforations.^25^, ^26^ In the article of Navarre et al., the abscesses detected during the surgical procedure spread to the ileocecal and even gluteal region in a large amount.^27^ They reported this situation as “impressive”.^27^ In *Actinomycosis* infections, the appendix has firmly fibrous adhesions and there are fibrotic tissues in the mesoappendix.^25^, ^28^, ^29^ The adhesions with *Actinomycosis* were defined as "woody adhesions" and the excision was "tedious".^29^ Liu et al. used the expressions "densely adherent" and "markedly thickened" for two patients with similar findings in their article.^30^ However, in the same study, appendicitis due to *Actinomycosis* was also reported as less than 1 cm in diameter.^30^ In two patients presented by Sung Y‐N et al., the findings were more extensive and ileocecal resection was required because ileocecal thickening was also accompanied.^31^ Schmidt et al. stated that the appearance of the appendix might be in the form of inflammatory pseudotumor.^26^

The literature of appendicitis due to *Actinomycosis* is very similar to the findings during our operation. We understand that by keeping this information in mind during the surgical procedure, surgeons may give priority to necessary microbiological procedures and pathological evaluations for *Actinomycosis*. We think this is one of the important points revealed by our research.

Some cases of appendicitis diagnosed with CD may have adhesions to the terminal ileum and cecal pouch.^3^, ^4^ It is remarkable for most appendicitis cases diagnosed with CD are defined as "firm".^3^, ^4^ In a case reported by Binder and Freeman, the surgeon was suspected of CD during surgery based on the enlarged and inflamed appearance of the appendix and it was stated that CD was finally diagnosed.^4^ The appearance in our patient is similar to that described for CD.

Therefore, in terms of the macroscopic appearance of our case, it is understood with the literature data that CD, as in *Actinomycosis*, can be considered primarily and Sarcoidosis may occur, albeit with a low probability.

The major part of the data we can obtain in the literature is in case presentations, clinical and histopathological studies that include the keywords "granulomatous” and “appendicitis". On the other hand, only some of these articles have data on the surgeon's evaluation made during surgery, and these evaluations are usually expressed in 1–2 short sentences in the “result” section. These expressions are underlined as "adhesion", "removal with difficulty" and "immobility of surrounding tissues" of the appearance of the granulomatous appendix.^11^, ^27^, ^29^ The literature data regarding the conversion of laparoscopic appendectomy to laparotomy may be meaningful in terms of finding information about the characteristics of granulomatous appendicitis. Since the most important factors for the surgeon to make a conversion decision are the prolongation of the period and the presence of a "severe" inflammation picture, as expected in granulomatous diseases.^8^, ^32^, ^33^, ^34^, ^35^, ^36^ However, in these studies, the degree of inflammation was evaluated with standard classification and was not expressed as indicating a specific disease.^36^ In a few of them, it has been reported that the risk of conversion is high in appendicitis surgeries involving "fibrotic adhesions".^32^, ^35^ In an article, inflammation related conversion is expressed as Grade 4–5.^32^ Still, it is unclear whether granulomatous diseases were also taken into account in this evaluation of inflammation classification.

The treatment of appendicitis due to granulomatous diseases may not always progress as expected during the appendectomy procedure. Unfortunately, it has been observed in the literature that the ability to evaluate granulomatous appendicitis during surgery is poorly reflected. The reason for this may be that such cases are not considered during operations because of their low incidence among appendicitis cases. Another possible reason may be the inability of the surgeons to contribute to the evaluation process by not providing information to the pathologist about the nature of the inflammation seen in the surgery. As can be understood from the literature, it is not enough to describe granulomatous diseases as inflammation only and to wait for histopathology result. If the surgeon can consider the pre‐diagnosis of granulomatous appendicitis during surgery, it will not be difficult to predict that the patient will save time in the diagnosis and treatment process. It is hard to say with one patient but my experience on this case prepare me to evaluate appendicitis cases for the possibility of a granulomatous disease if preoperative symptoms, findings and physical examination are showing different directions that confuses surgeon. Also, appendix with “woody adhesions” and “inflammatory appearance without fragile and enormous vascularize tissue must cause awareness about granulomatous disease.

## 3 CONCLUSION

When a granulomatous disease is diagnosed in the appendix, the treatment will differ from the standard appendicitis treatment. Therefore, it should be considered that making a surgical opinion about diseases with a possibility of granulomatous disease would be beneficial for the patient. Unfortunately, there is no classification in which inflammation and histologic findings determined by the surgeons are evaluated together. All of the current definitions in literature are to predict the possibility of complications. Moreover, as can be understood from the literature that surgeons do not pay enough attention for granulomatous diseases in the macroscopic evaluation of appendicular inflammation during surgery. It can be said that the definition of appendix inflammation made by the surgeon should not only be in terms of predicting complications. Literature on the subject should be increased.

## CONFLICT OF INTEREST

There is no conflict of interest.

## AUTHOR CONTRIBUTION

The author prepared all manuscript.

## ETHICAL APPROVAL

Ethical approval is not needed for this case report.

## 4 CONSENT

Written informed consent is taken from the patients’ parents according to the journal policy.

## Data Availability

The data that support the findings of this study are available from the corresponding author upon reasonable request.

## References

[1] ‐ Andrews EW . Tuberculosis Herniosa and Appendicitis (Presentation) Chicago Surgical Society, I901: 787‐795.

[2] Otsukaa R , Shinotoa K , Okazakia Y , et al. Crohn’s Disease Presenting as Granulomatous Appendicitis. Case Rep Gastroenterol. 2019;13(3):398‐402. 31616234 10.1159/000503170 PMC6792421

[3] Allen DC , Biggart JD . Granulomatous disease in the vermiform appendix. J Clin Pathol. 1983;36(6):632‐638. 6853730 10.1136/jcp.36.6.632 PMC498340

[4] Binder H , Freeman H , Granulornatous (Crohn's) appendicitis. Can J Gastroenterol. 1991; 3:112‐117.

[5] Constantinescu C , Vayalumkal J , Fisher D . An unusual case of appendicitis. CMAJ. 2014;186(16):1241‐1243. 25096665 10.1503/cmaj.131126 PMC4216258

[6] Wu H , Fuller MY , Pogoriler J . Granulomatous Appendicitis in the Pediatric Population. Pediatr Dev Pathol. 2020;23(3):215‐221. 31619135 10.1177/1093526619881545

[7] Charfi S , Sellami A , Affes A , Yaïch K , Mzali R , Boudawara TS . Histopathological findings in appendectomy specimens: a study of 24,697 cases. Int J Colorectal Dis. 2014;29(8):1009‐1012. 10.1007/s00384-014-1934-7. 24986137

[8] Shindholimath VV , Thinakaran K , Rao NT , Veerappa Y . Laparoscopic management of appendicular mass. Journal of Minimal Access Surgery. 2011;7(2):136–140. 10.4103/0972-9941.78345. 21523236 PMC3078476

[9] Pei H , Law WL , Choy C , Chan GSW , Chu KW . Granulomatous Appendicitis Progressing to Crohn’s Disease with Bleeding Complication. ANZ J Surg. 2003;73 (7):554‐556. 12864840 10.1046/j.1445-1433.2003.02663.x

[10] Rabah R . Pathology of the appendix in children: an institutional experience and review of the literature. Pediatr Radiol. 2007;37(1):15‐20. 10.1007/s00247-006-0288-x. 17031635

[11] Pal K . Granulomatous appendicitis in children: A single institutional experience. Afr J Paediatr Surg. 2014;11(1):26‐31. 10.4103/0189-6725.129207. 24647289

[12] Bolmers MDM , de Jonge J , van Rossem CC . Discrepancies between Intraoperative and Histological Evaluation of the Appendix in Acute Appendicitis. Journal of Gastrointestinal Surgery. 2020;24(9):2088–2095. 10.1007/s11605-019-04345-3. 31410818

[13] van den Boom Al , de Wijkerslooth Eml , Mauff Kal , Interobserver variability in the classification of appendicitis during laparoscopy. BJS. 2018;105(8):1014–1019. 10.1002/bjs.10837. PMC6033013 29663311

[14] Pham H , Devadas M , Howle J . Effect of surgical experience on the macroscopic diagnosis of appendicitis: A retrospective cohort study. Int J Surg. 2015;16:78e82. 25749438 10.1016/j.ijsu.2015.02.019

[15] Hamminga JTH , Hofker HS , Broens PMA , Kluin PM , Heineman E , Haveman JW . Evaluation of the appendix during diagnostic laparoscopy, the laparoscopic appendicitis score: a pilot study. Surg Endosc. 2013;27(5):1594–1600. 23073690 10.1007/s00464-012-2634-4

[16] Rodríguez E , Valero J , Jaramillo L , Vallejo‐Ortega MT , Lagos L . Evaluation of concordance among surgeons and pathologists regarding the diagnosis and classification of acute appendicitis in children. J Pediatr Surg. 2020;55(8):1503‐1506. 31718870 10.1016/j.jpedsurg.2019.09.025

[17] Sadot E , Keidar A , Shapiro R , Wasserberg N . Laparoscopic accuracy in prediction of appendiceal pathology: oncologic and inflammatory aspects. The American Journal of Surgery. 2013;206(5):805‐809. 24016703 10.1016/j.amjsurg.2013.05.002

[18] So‐Hyun N , Jin SK , Ki HK , Sung JP . Primary tuberculosis appendicitis with mesenteric mass. J Korean Surg Soc. 2012;82(4):266‐269. 22493770 10.4174/jkss.2012.82.4.266 PMC3319783

[19] Chandra BJS , Girish TU , Thrishuli PB , Vinay HG . Primary Tuberculosis of the Appendix: A Rare Cause of a Common Disease. Journal of Surgical Technique and Case Report. 2013;5(1):32‐34. 24470848 10.4103/2006-8808.118619 PMC3889001

[20] Rabenandrasana HA , Ahmad A , Samison LH , Vololonantenaina C , Andrianandrasana A , Murata K . Child primary tubercular appendicitis. Pediatr Int. 2004;46(3):374‐376. 15151563 10.1111/j.1442-200x.2004.01904.x

[21] Rohena F , Almira‐Suarez MI , Gonzalez‐Keelan C . Granulomatous Enterocolitis Secondary to Yersinia in an 11‐year‐old Boy from Puerto Rico, Confirmed by PCR: A Case Report. P R Health Sci J. 2014;33:27‐30. 24665606

[22] Iida T , Yamashita K , Arimura Y , Shinomura Y . Colonic Sarcoidosis Presenting as Granulomatous Appendicitis. J Gastrointestin Liver Dis. 2016;25(1):8. 27014748 10.15403/jgld.2014.1121.251.csa

[23] Erra P , Crusco S , Nugnes L , et al. Colonic sarcoidosis: Unusual onset of a systemic disease. World J Gastroenterol. 2015;21(11):3380‐3387. 25805948 10.3748/wjg.v21.i11.3380 PMC4363771

[24] Karagülle E , Turan H , Turk E , Kiyici H , Yildirim E , Moray G . Abdominal actinomycosis mimicking acute appendicitis. Can J Surg. 2008;5(15):E109‐E110. PMC2556537 18841226

[25] Gomez‐Torres GA , Ortega‐Garcia OS , Gutierrez‐Lopez EG , et al. A Rare Case of Subacute Appendicitis, actinomycosis as the final pathology report: A case report and literature review. Int J Surg Case Reports. 2017;36:46‐49. 10.1016/j.ijscr.2017.04.033 PMC5440354 28531869

[26] Schmidt P , Koltai JL , Weltzien A . Actinomycosis of the appendix in childhood. Pediatr Surg Int. 1999;15(1):63‐65. 9914360 10.1007/s003830050515

[27] Navarrea P , Cantina MA , Isler MH . Para‐iliac actinomycetoma presenting as sarcoma, a late complication of appendicitis: A case report. Int J Surg Case Reports. 2014;5(2):43‐46. 10.1016/j.ijscr.2013.11.002 PMC3921643 24434727

[28] Nissotakis C , Sakorafas GH , Koureta T , Revelos K , Kassaras G , Peros G . Actinomycosis of the appendix: diagnostic and therapeutic considerations. IJID. 2008;12(5):562–564. 10.1016/j.ijid.2007.12.015. 18400543

[29] Liu V , Val S , Kang K , Velcek F . Case report: actinomycosis of the appendix—an unusual cause of acute appendicitis in children. J Pediatr Surg. 2010;45(10):2050‐2052. 20920728 10.1016/j.jpedsurg.2010.06.015

[30] Liu K , Joseph D , Lai K , Kench J , Ngu MC . Abdominal actinomycosis prtwo case reports and review. Journal of Surgical Case Reports. 2016;5:1‐3. 10.1093/jscr/rjw068 PMC4855211 27147718

[31] ‐ You‐Na S , Jihun K . Appendiceal actinomycosis mimicking appendiceal tumor, appendicitis or infammatory bowel disease. ournal of Pathology and Translational Medicine 2020; 26: 1‐6. 10.4132/jptm.2020.05.17 PMC8476320 32580538

[32] Wagner PL , Eachempati SR , Aronova A , et al. Contemporary Predictors of Conversion from Laparoscopic to Open Appendectomy. Surgical Infections. 2011;12(4):261‐266. 21790480 10.1089/sur.2010.079

[33] Metzelder ML , Jesch N , Dick A , Kuebler J , Petersen C , Ure BM . Impact of prior surgery on the feasibility of laparoscopic surgery for children: a prospective study. Surg Endosc. 2006;20(11):1733‐1737. 10.1007/s00464-005-0772-7. 17024536

[34] Sakpal SV , Bindra SS , Chamberlain RS . Laparoscopic Appendectomy Conversion Rates Two Decades Later: An Analysis of Surgeon and Patient‐Specific Factors Resulting in Open Conversion. J Surg Res. 2012;176(1):42‐49. 21962732 10.1016/j.jss.2011.07.019

[35] Kostov K . Acute appendicitis‐laparoscopic appendectomy and reasons for conversion. Journal of IMAB ‐ Annual Proceeding (Scientific Papers). 2020;26(1):2991–2993.

[36] Antonacci N , Ricci C , Taffurelli G , et al. Laparoscopic appendectomy: Which factors are predictors of conversion? A high‐volume prospective cohort study. Int J Surg. 2015;21:103‐107. 26231996 10.1016/j.ijsu.2015.06.089

